# Neural Correlates and Adaptive Mechanisms in Vascular Cognitive Impairment: Exploration of a Structure–Function Coupling Network

**DOI:** 10.1111/cns.70205

**Published:** 2025-03-09

**Authors:** Jing Jin, Jie Ma, Jia‐Jia Wu, Juan‐Juan Lu, Hao‐Yu Lu, Mou‐Xiong Zheng, Xu‐Yun Hua, Jian‐Guang Xu

**Affiliations:** ^1^ School of Rehabilitation Science Shanghai University of Traditional Chinese Medicine Shanghai China; ^2^ Department of Rehabilitation Medicine Yueyang Hospital of Integrated Traditional Chinese and Western Medicine, Shanghai University of Traditional Chinese Medicine Shanghai China; ^3^ Department of Traumatology and Orthopedics Shuguang Hospital, Shanghai University of Traditional Chinese Medicine Shanghai China; ^4^ Engineering Research Center of Traditional Chinese Medicine Intelligent Rehabilitation Ministry of Education Shanghai China

**Keywords:** function network, structure network, structure–function coupling, vascular cognitive impairment, white matter hyperintensity

## Abstract

**Background:**

Cerebral small vessel disease exacerbates cognitive decline, yet the structural–functional coupling mechanisms in vascular cognitive impairment (VCI) remain unclear.

**Methods:**

This study included 121 participants, with 68 individuals with VCI and 53 healthy controls. Participants underwent neuropsychological assessments and multimodal imaging. We compared white matter integrity, structural network topology, and functional network topology between groups, exploring the relationship between structure–function coupling and cognitive function. Family‐wise error (FWE) correction was applied to account for multiple comparisons.

**Results:**

VCI participants showed reduced fractional anisotropy and increased mean and radial diffusivity in white matter. Structural network analysis revealed lower global and local efficiency, reduced small‐world properties, and increased characteristic path length. Nodal properties, particularly in key regions of the default mode and visual networks, were significantly altered in VCI participants. While no significant differences were observed in functional network topology, VCI participants exhibited enhanced structure–function coupling in critical nodes of the default mode and visual networks. This enhancement correlated with memory function and information processing speed in the temporal calcarine, insula, occipital, and lingual regions.

**Conclusions:**

The study identifies disrupted brain networks and enhanced compensatory mechanisms in VCI, offering insights into neuroplasticity in VCI and contributing to dementia prevention strategies.

## Graphical abstract

Our study revealed significant disruptions in brain structural networks in vascular cognitive impairment participants, including reduced fractional anisotropy and altered network properties. Despite no significant functional topology differences, enhanced structure–function coupling in key regions correlated with cognitive performance, suggesting compensatory neuroplasticity and offering insights for cognitive disorders prevention.

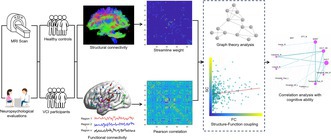

## 1 Introduction

Cerebrovascular pathology contributes to the progression of dementia, often synergizing with neurodegenerative processes to exacerbate cognitive decline and functional impairment [1]. Vascular cognitive impairment (VCI), encompassing all cognitive aberrations associated with vascular pathology, constitutes approximately 20%–40% of all dementia diagnoses, highlighting its significant role in cognitive decline [2, 3]. As a growing public health concern, age‐related cognitive decline places a significant economic burden on society, especially considering the escalating prevalence of dementia with aging populations worldwide.

VCI is generally stated to be the second most common cause of dementia in later life in Caucasian populations and may be the most common cause in East Asia. There are few data from other ethnic groups [4]. White matter hyperintensities (WMH), a hallmark imaging feature of cerebral small‐vessel disease (CSVD), are indicative of microangiopathic axonal damage and demyelination, often seen in regions of white matter degeneration [5, 6]. These lesions are not only reflective of structural damage but are also increasingly recognized for their association with cognitive deficits in VCI. Effective prevention and intervention strategies for dementia necessitate a comprehensive understanding of the neuroplasticity mechanisms underlying VCI, as these insights could direct novel therapeutic approaches.

Evidence indicates that patients with WMH have a 14% increased risk of cognitive impairment and all‐cause dementia [7, 8]. The extent and location of WMH are associated with the severity of cognitive decline, as well as an increased risk of Alzheimer's disease and VCI. Studies have found that overall cortical thickness and medial temporal lobe thickness mediate the relationship between WMH volume, overall cognition, and memory function [9]. Mayer et al. [10] measured cortical thickness on MRI images and segmented WMH on FLAIR images in a community‐based sample of 930 individuals, finding that periventricular WMH were associated with reduced cortical thickness. In addition to their role in maintaining structural integrity, recent studies suggest that WMH may also disrupt the brain's functional networks. These disruptions have been shown to correlate with cognitive decline, particularly in information processing speed, executive function, attention, and memory [11]. The mechanisms by which WMH affect VCI likely involve chronic ischemia, neuroinflammation, venous collagen deposition leading to reduced cerebral blood flow, and blood–brain barrier dysfunction, all contributing to a gradual loss of neuronal integrity and connectivity in the brain [12, 13, 14]. In summary, the association between WMH and VCI underscores WMH's potential role as a significant contributor to the development and progression of VCI and other age‐related cognitive disorders.

The impact of brain functional and structural remodeling on cognitive abilities has been substantiated [15]. VCI, arising from structural disruption due to cerebrovascular disease, leads to cognitive dysfunction. While structural and functional connectedness are associated, substantial functional connections can exist between regions without direct structural relationships [16]. Investigating the coupling between brain function and structure is pivotal for elucidating the central mechanisms underlying cognitive impairment in VCI, an area that has yet to be thoroughly explored in research. Studies have shown disrupted brain connectivity in VCI individuals, including functional networks constructed from functional magnetic resonance imaging (fMRI) [17] and structural networks from diffusion tensor imaging (DTI) [18, 19]. Functional and structural coupling is a new integrative measure linking the functional and structural networks. It is regarded as a more sensitive method for detecting slight changes in brain activity than any single‐modality index [20]. However, discrepancies between the results of functional and structural connectivity analyses present a challenge in selecting appropriate therapeutic targets. Therefore, investigating the coupling between structural and functional networks in VCI to identify these differences is crucial.

In this study, we hypothesized that (1) participants with VCI exhibit lower fiber tract integrity and less efficient structural network connectivity, (2) participants with VCI will show a decline in functional network connectivity, based on decreased structural network communication efficiency, and (3) participants with VCI will exhibit differential structural–functional network coupling in cognitive‐related brain regions. To test these hypotheses, we assessed participants using fMRI and diffusion MRI scans, along with comprehensive neuropsychological assessments. Initially, we conducted baseline statistical analyses of the clinical characteristics and cognitive levels of VCI participants and healthy controls (HCs). We then utilized tract‐based spatial statistics (TBSS) and network topology analyses on the MRI data to explore differences in functional and structural coupling between VCI participants and HCs.

## 2 Materials and Methods

### 2.1 Participants

This was a case–control study involving 121 right‐handed participants. The protocol of this study was approved by the Medical Ethics Committee of Yueyang Hospital of Integrated Traditional Chinese and Western Medicine, Shanghai University of Traditional Chinese Medicine. At baseline, all participants underwent a neuropsychological evaluation and MRI conducted by professional imaging staff. They were also provided with information about the study and were required to sign informed permission forms.

The trail inclusion criteria were as follows: (i) 50 < age < 75; (ii) Summary Mental State Examination (MMSE) score ≥ 24; (iii) Hamilton Depression Scale (HAMD) score scale < 8 points; (iv) Modified Rankin Scale (MRS) score of ≤ 1; and (v) Hamilton Anxiety Scale (HAMA) rating scale < 7 points; (vi) right‐handedness; (vii) National Institutes of Health Stroke Scale (NIHSS) score < 2. For VCI participants, they must meet the diagnostic criteria as defined by consensus guidelines [21], provide objective evidence of cognitive impairment or decline, characterized by at least 1.0 standard deviation below age‐ and education‐adjusted norms in any cognitive domain, and have white matter lesions, as evidenced by Fazekas scores of 1 or greater. HCs must show no signs of cognitive impairment in any cognitive domain and must not have any white matter lesions, as indicated by a Fazekas score of 0 [22]. Exclusion criteria were as follows: (i) MRI indicating other brain parenchymal lesions (e.g., malformation or tumor); (ii) history of other neurological or psychiatric disorders (e.g., Alzheimer's disease, epilepsy, Parkinson's disease, ans schizophrenia); (iii) severe cardiac, hepatic, or renal insufficiency; (iv) inability to complete neuropsychological assessments; (v) inability to undergo brain MRI.

### 2.2 Neuropsychological Assessments

Neuropsychological assessments were conducted by a neuropsychologist who was blinded to the clinical characteristics of the participants. A second neuropsychologist, also blinded to the clinical details, performed the scoring of the assessments. The neuropsychological assessment included the Mini‐Mental Status Examination (MMSE) scores [23] and performance in four cognition domains: (1) episodic memory: Auditory Verbal Learning test (AVLT) [24, 25], (2) working memory: Trail Making test (TMT) (B time) [26], (3) attention: Symbol Digit Modalities test (SDMT), [27] and (4) language tests: Boston Naming Test (BNT) [28].

### 2.3 Fazekas Score Evaluation

The WMH grade was assessed using the Fazekas classification method on T2‐weighted fluid‐attenuated inversion recovery imaging (FLAIR). PVWMH and DWMH were scored separately and summed for the total Fazekas score. PVWMH scores: 0 (none), 1 (caps/pencil‐thin lining), 2 (smooth halo), and 3 (irregular). DWMH scores: 0 (none), 1 (punctate), 2 (beginning confluence), and 3 (large confluent). Total scores: mild (1, 2), moderate (3, 4), and severe (5, 6). This procedure was evaluated by two neurologists who were unaware of the participants' clinical features. In cases of discrepancies in MRI interpretations, a third neurologist reviewed the images to reach a consensus. This double‐blind methodology ensured the objectivity and reliability of the neuropsychological evaluations and imaging analyses.

### 2.4 MRI Acquisition

MRI data were acquired using a Siemens MAGNETOM Verio, a high‐field 3.0 Tesla scanner manufactured in Erlangen, Germany. Participants were guided to keep their eyes shut, remain tranquil, and avoid dozing off throughout the MRI scan. Resting‐state functional MRI images were captured through a gradient‐echo echo‐planar imaging sequence with the following specifications: axial orientation; repetition interval of 3000 ms; field of view measuring 203 mm × 203 mm; matrix dimensions of 64 × 64; flip angle set at 52°; slice thickness of 3.0 mm; 43 slices; no gap (voxel size equates 3.6 mm × 3.6 mm by 3.0 mm); and a total of 200 volumes acquired. The diffusion‐weighted imaging dataset was collected with these parameters: TR at 10,000 ms, TE at 89 ms, flip angle at 90°, matrix size of 128 × 128, FOV of 240 × 240 mm, slice thickness of 2.0 mm, and resolution of 1.875 × 1.875 × 2.0 mm. The DWI series includes 60 diffusion‐weighted images taken across 60 non‐collinear gradient directions with a b‐value of 1000 s/mm^2^, complemented by two additional images with a b‐value of 0 s/mm^2^. High‐resolution T1‐weighted structural images were acquired using a 3D magnetization‐prepared rapid gradient‐echo (T1WI‐3D‐MPRAGE) sequence. The scanning parameters were as follows: TR = 1900 ms, TE = 2.93 ms, flip angle = 9°, matrix = 256 × 256, FOV = 256 mm × 256 mm, slice thickness = 1.0 mm, and resolution = 1 × 1 × 1 mm.

### 2.5 Preprocessing

Details of MRI data preprocessing can be found in the Supporting Informations.

### 2.6 Spatial Statistics Along White Matter Tracts

A voxel‐based analysis using TBSS was applied to evaluate fractional anisotropy (FA), mean diffusivity (MD), axial diffusivity (AD), and radial diffusivity (RD) metrics. The methodology consisted of several steps: (i) registration of individual FA images to the MNI standard space using linear and nonlinear techniques [29]; (ii) creation of a mean FA template and a fiber skeleton thresholded at 0.2 [29]; (iii) projection of aligned FA images onto the mean FA skeleton for voxel‐wise analysis; (iv) application of TBSS to MD, AD, and RD data using the FSL software suite [30]; and (v) extraction of mean FA, MD, AD, and RD values for each tract in the Johns Hopkins University (JHU) White Matter Tractography Atlas [31] for group comparisons.

### 2.7 Structural Network Construction and Graph Theoretical Analysis

The individual FA skeletons constructed as described above were used to build structural brain networks. The structural connectivity network was modeled as an unweighted network comprising 90 nodes, defined by the automated anatomic labeling (AAL) template [32]. The fiber assignment by continuous tracking (FACT) algorithm was employed to track white matter fibers between node pairs [33]. Fiber tracking was terminated when the FA value dipped below 0.2 or the tracking angle surpassed 45° [29, 34]. The connectivity within the brain network was quantified by the count of fibers linking each pair of regions, culminating in a 90 × 90 network matrix. The binary threshold of retaining connections in over 80% of participants was chosen to ensure reliable connections. By focusing on connections present in the majority, we reduce the impact of individual variability and noise, highlighting more stable and biologically meaningful connections [35]. If the fiber count between nodes i and j was 3 or more, a binary value of 1 was designated for the connection $\left(i,j\right)$ element $\left(e_{j,j}\right)$. Conversely, if the count was less than 3, $e_{j,j}$ was set to 0. This thresholding method significantly minimizes the risk of spurious connections due to noise or limitations inherent in deterministic tractography [36]. Consequently, a binary 90 × 90 matrix was assembled, representing the structural connectivity of the white matter in the brain. Subsequent binary graph theory‐based network analyses were performed in the GRETNA toolbox [37]. Several key graph measures were calculated, including global efficiency (Eglob), local efficiency (Eloc), shortest path length (Lp), clustering coefficient (Cp), small‐world parameters (Sigma), nodal efficiency (NEglob), nodal local efficiency (NEloc), betweenness centrality, degree centrality, nodal clustering coefficient (NCp), and nodal shortest path length (NLp). The definition and calculation formula can be found in the Supporting Informations.

### 2.8 Functional Network Construction and Graph Theoretical Analysis

Following the de‐noising process, the functional connectivity between each pair of brain regions was measured by computing the Pearson correlation coefficient between the average BOLD time series of the regions. A 90 × 90 weighted adjacency matrix, representing the connectome, was then created for each participant. Fisher Z‐transformation was used to increase the normal distribution properties of the network. The sparsity threshold range was set from 0.01 to 0.40 with a step size of 0.01 to generate binary undirected networks characterized by small‐world architecture and connectivity. Based on the constructed functional network matrix, GRETNA was also used to calculate the topological properties of the functional network, including global efficiency (Eglob), local efficiency (Eloc) shortest path length (Lp), clustering coefficient (Cp), small‐world parameters (Sigma), nodal efficiency (NEglob), nodal local efficiency (NEloc), betweenness centrality, degree centrality, nodal clustering coefficient (NCp), and nodal shortest path length (NLp).

### 2.9 Structure–Function Coupling Analysis

The regional connectivity signatures were derived from individual columns of each participant's structural or functional connectivity matrix, encapsulated as vectors that quantify the strength of connections emanating from a single network node to the remainder of the nodes. The coupling between the structure and function was quantified using the Spearman rank correlation applied to the non‐zero components of both structural and functional connectivity profiles [38, 39]. These regional measures of structure–function coupling were subsequently averaged across the cohort of participants.

### 2.10 Numerical Data Statistical Analysis

Data were analyzed using SPSS 25.0 software (IBM, Armonk, NY, USA). The Shapiro–Wilk test was employed to assess the normality of numerical data; if the *p* value from the test was greater than 0.05, the data were considered normally distributed. For MRI image data, Fisher's Z‐transformation was applied to enhance the normality of the data. Normally distributed continuous variables were analyzed with the independent t‐test, with results presented as mean ± standard deviation. For non‐normally distributed continuous variables, the Mann–Whitney U test was used, and the results were expressed as median and interquartile range. Categorical variables were expressed as percentages and compared using the chi‐squared test. Pearson's correlation analysis was applied for normally distributed data, while Spearman's correlation analysis was used for non‐normally distributed or categorical data. In the group comparison process, linear regression was employed to control for the effects of age, gender, and education level on statistical outcomes. Following this regression of covariates, partial correlation analysis was conducted to explore the relationship between brain network attributes and cognitive performance. All hypothesis tests were two‐tailed, and *p* values < 0.05 were considered significant. Family‐wise error (FWE) correction was applied to reduce errors from multiple comparisons.

## 3 Results

### 3.1 Participants

**FIGURE 1:**
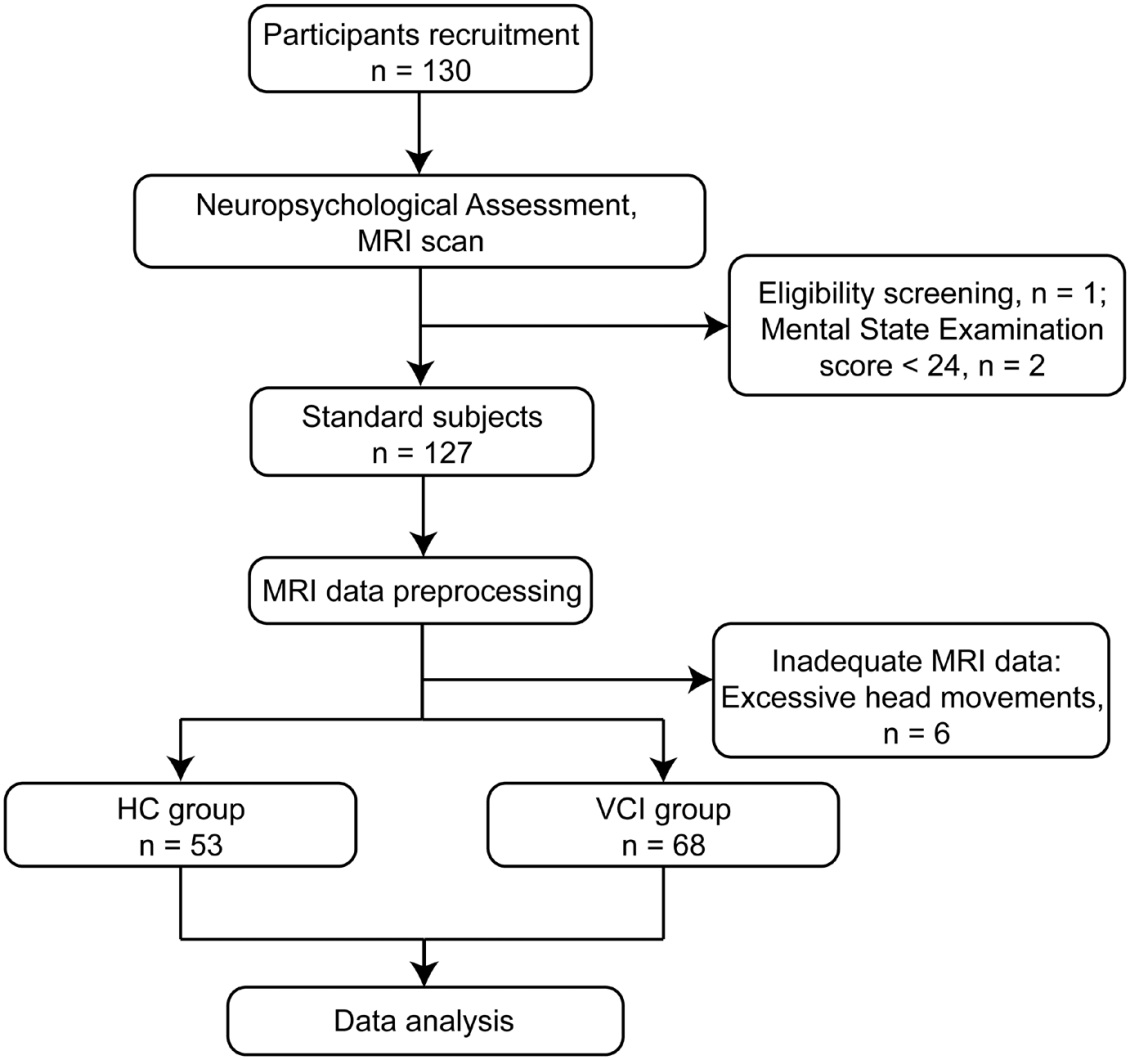
Enrollment and disposition of participants. Abbreviations: HC, healthy control; MRI, magnetic resonance imaging; N, number; VCI, vascular cognitive impairment.

A total of 130 subjects were recruited into the study at first. All participants underwent neuropsychological assessment and MRI scanning, including DTI and BOLD images. We visually inspect the MRI images of the subjects and exclude those with excessive artifacts. One subject with severe MRI imaging artifacts and two subjects with MMSE scores below 24 were excluded after MRI evaluation. The images of participants whose head movements exceeded 3.0 mm or whose rotations were over 3.0° were considered to have an impact on the analysis results and thus were excluded. Following image preprocessing, six subjects with excessive head movements were further excluded, resulting in 121 subjects included in the final analysis. Subjects were categorized into HCs (*n* = 53) versus VCI participants (*n* = 68) based on cognitive scales and Fazekas scores. Finally, the data were analyzed statistically (Figure 1).

Participants' baseline demographic characteristics were comparable between the two trial groups. The VCI group exhibited inferior cognitive performance across memory, executive function, information processing speed, attention, and naming ability. Detailed information is available in Table 1.

**TABLE 1:** Baseline demographic and clinical characteristics of participants.

Characteristics	Total	HC group	VCI group	F/Z/X^2^	*p* ^a^
	(*n* = 121)	(*n* = 53)	(*n* = 68)		
Gender, male *n* (%)	53 (43.80)	20 (37.74)	33 (48.53)	3.324	0.068
Age, yrs	68 ± 5.94	66.81 ± 5.35	68.76 ± 6.24	2.285	0.072
Height, cm	163.24 ± 7.42	164.11 ± 6.54	162.56 ± 8.02	1.941	0.255
Weight, kg	62.50 ± 9.70	62.25 ± 8.55	63.65 ± 10.54	2.321	0.435
Education, yrs	10 (9,12)	11 (9,14)	9 (9,12)	−1.612	0.107
Smoke, *n* (%)	29 (23.97)	7 (13.20)	13 (19.12)	0.754	0.385
Drink, *n* (%)	16 (13.22)	5 (9.43)	11 (16.18)	1.180	0.277
Hypertension, *n* (%)	61 (50.41)	23 (43.40)	38 (55.88)	1.858	0.173
Hypercholesterolemia, *n* (%)	38 (31.40)	12 (22.64)	26 (38.24)	3.362	0.067
Diabetes, *n* (%)	26 (21.49)	9 (16.98)	17 (25.00)	1.135	0.287
MMSE	28 (26,29)	28 (27,29)	27 (25,28)	−4.338	< 0.001***
AVLT	27.05 ± 9.33	31.45 ± 8.81	21.19 ± 7.00	1.450	< 0.001***
AVLT_N5	5 (3,6)	6 (5,7)	3 (2,5)	−5.965	< 0.001***
SDMT	32.69 ± 13.26	40.25 ± 12.24	26.79 ± 10.87	0.025	< 0.001***
STT_B	134 (105,190)	114 (98,138)	165 (119,238)	4.757	< 0.001***
BNT	23 (21,26)	25 (23,26)	21.99 ± 3.44	−3.800	< 0.001***
HAMD	0 (0,2)	1 (0,2)	0 (0,1)	−1.355	0.176
HAMA	2 (1,4)	2 (1, 4.5)	2 (1,3)	−0.852	0.394

*Note:* alues are mean ± SD, *n* (%) or median (interquartile range). ^a^p‐value for between‐group comparison significance; ***, a statistically significant difference was observed between vascular cognitive impairment (VCI) participants and healthy controls (HCs).

Abbreviations: AVLT, Auditory Verbal Learning Test; AVLT_N5, Auditory Verbal Learning Test, Long‐Term Delay Recall; BNT, Boston Naming Test; HAMA, Hamilton Anxiety Scale; HAMD, Hamilton Depression Rating Scale; MMSE, Mini‐Mental State Examination; SDMT correct, Symbol Digit Modalities Test, correct number; STT_B, Shape Trails Test, part B.

### 3.2 Variability of Diffusion Tensor Metrics in VCI Individuals

**FIGURE 2:**
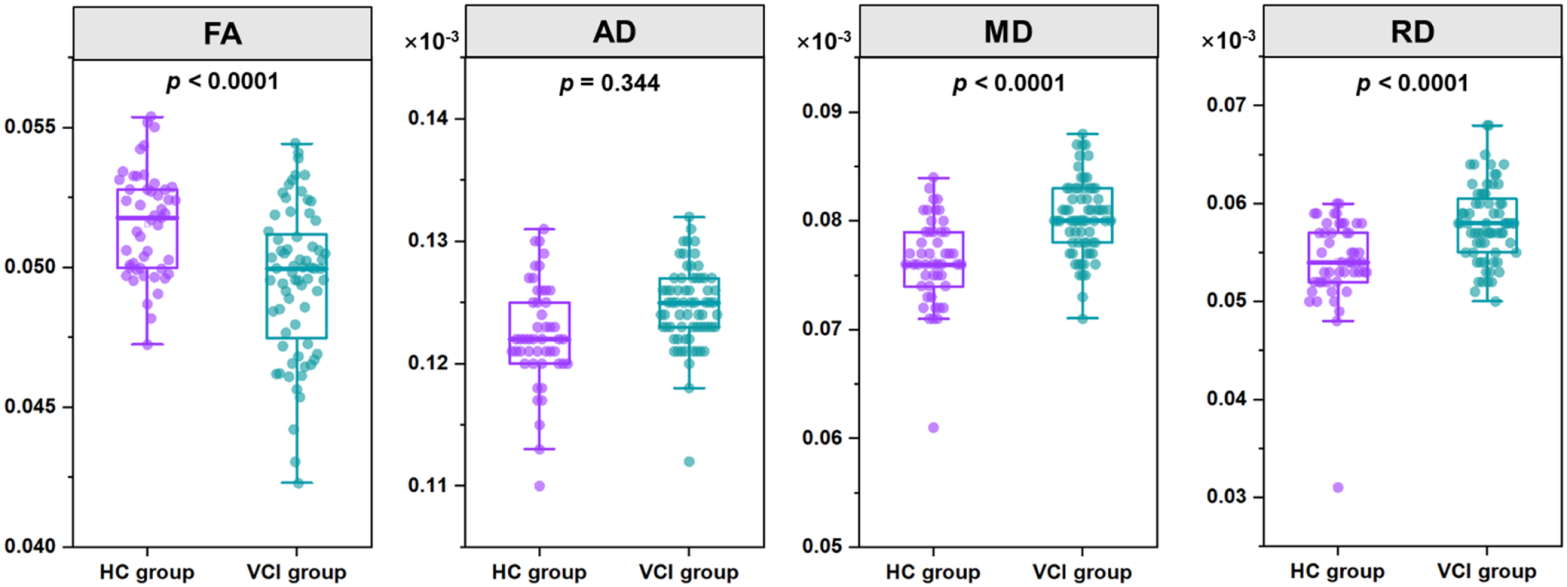
Group differences of diffusion tensor metrics for comparison between vascular cognitive impairment (VCI) participants and healthy controls (HCs). Abbreviations: AD: axial diffusivity; FA: fractional anisotropy; MD: mean diffusivity; RD: radial diffusivity.

In comparison to the HCs, VCI participants exhibited significant damage to their whole‐brain white matter fiber tracts. To quantify these changes, FA, MD, AD, and RD values were extracted and subjected to statistical analysis between the groups. The results revealed that, compared to HCs, FA values were significantly decreased in VCI participants. Conversely, MD and RD values were significantly increased in VCI participants, indicating compromised white matter integrity (Figure 2).

### 3.3 Structural Network Connectivity

**FIGURE 3:**
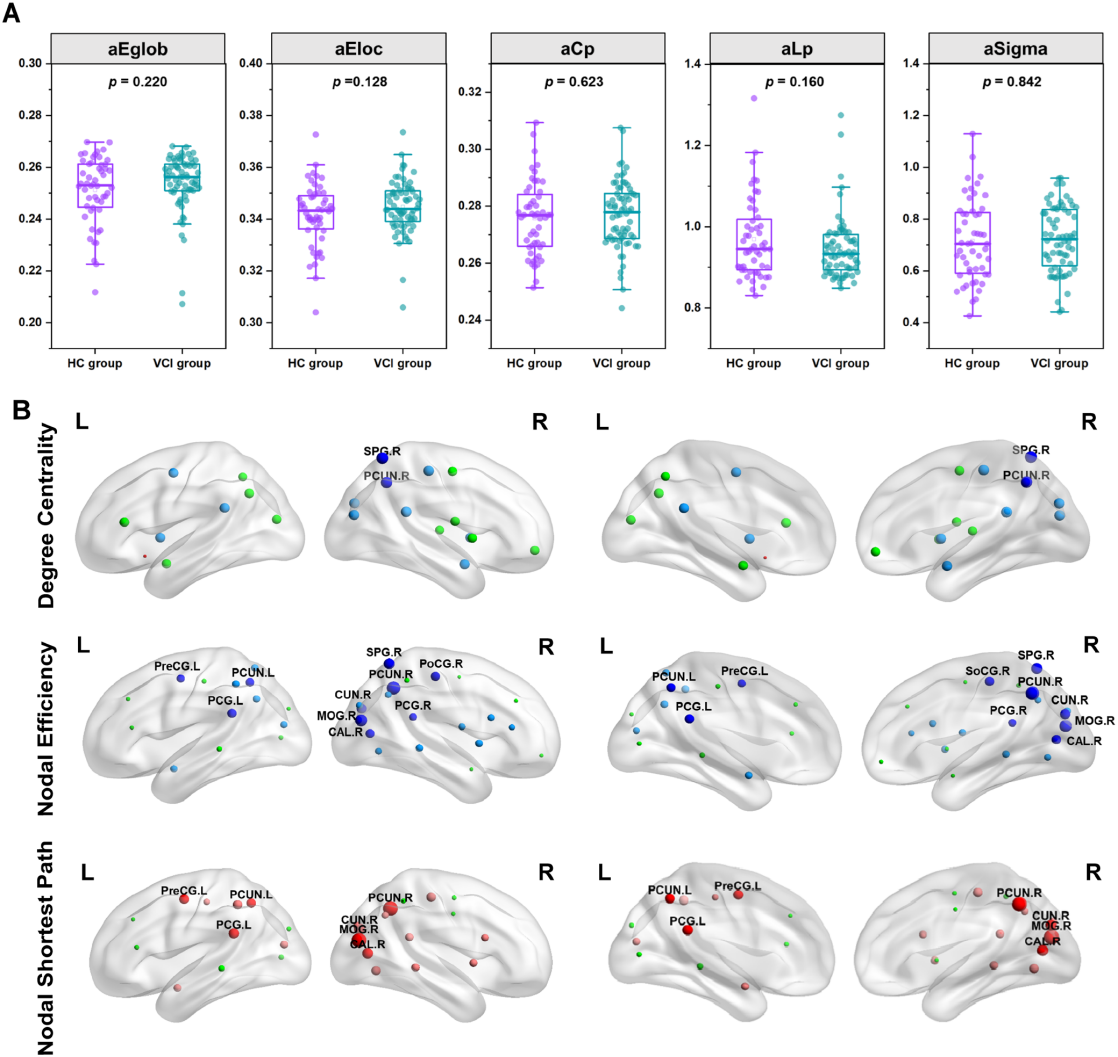
Group differences in structural network topological properties between vascular cognitive impairment (VCI) participants and healthy controls (HCs). (A) Box‐and‐whisker plots illustrating the area under the curve (AUC) parameters of global efficiency (Eglob), local efficiency (Eloc), clustering coefficient (Cp), characteristic path length (Lp), and small‐worldness (Sigma) for VCI participants and healthy controls. Means and standard deviations are depicted. (B) Group differences in degree, efficiency, and shortest path at the nodal level. Insignificant nodes are shown as green spheres, whereas blue (VCI<HC) and red (VCI>HC) spheres denote significant differences after FWE correction, with regions labeled where *p* < 0.001. The size of the significant nodes reflects the effect sizes of group differences. Abbreviations: AMYG, amygdala; CAL, calcarine cortex; CUN, cuneus; L, left; MOG, middle occipital gyrus; PCG, posterior cingulate gyrus; PCUN, precuneus; PoCG, postcentral gyrus; PreCG, precentral gyrus; PUT, putamen; R, right; SPG, superior parietal gyrus.

In analysis of structural network topological properties, participants with VCI showed lower global structural connectivity compared to HCs, specifically with reduced global efficiency (*t* = 2.789, *p* = 0.006), lower local efficiency (*t* = 2.528, *p* = 0.013), diminished small‐world properties (*t* = 2.642, *p* = 0.009), and increased characteristic path length (*t* = −2.693, *p* = 0.008) (Figure 3A).

As for nodal properties, VCI participants exhibited statistically significant decreases in the degree centrality of the left posterior cingulate gyrus, right precuneus, right posterior cingulate gyrus, right middle occipital gyrus, right cuneus, right superior parietal gyrus, right postcentral gyrus, right putamen, and right amygdala, as well as the left putamen, compared to HCs (*p* < 0.05, FWE‐corrected). Participants with VCI had a decreased nodal efficiency in left posterior cingulate gyrus (PCG.L), right precuneus (PCUN.R), left precuneus (PCUN.L), right posterior cingulate gyrus (PCG.R), right middle occipital gyrus (MOG.R), right cuneus (CUN.R), right calcarine cortex (CAL.R), right superior parietal gyrus (SPG.R), right postcentral gyrus (PoCG.R), and left precentral gyrus (PreCG.L) (*p* < 0.05, FWE‐corrected). We also found increased nodal shortest path in left posterior cingulate gyrus (PCG.L), right precuneus (PCUN.R), left precuneus (PCUN.L), right middle occipital gyrus (MOG.R), right cuneus (CUN.R), right calcarine cortex (CAL.R), and left precentral gyrus (PreCG.L) in VCI participants compared to that in HCs (*p* < 0.05, FWE‐corrected) (Figure 3B). No significant differences in betweenness centrality, nodal local efficiency, or nodal clustering coefficient were found between participants with VCI and HCs.

### 3.4 Functional Network Connectivity

**FIGURE 4:**
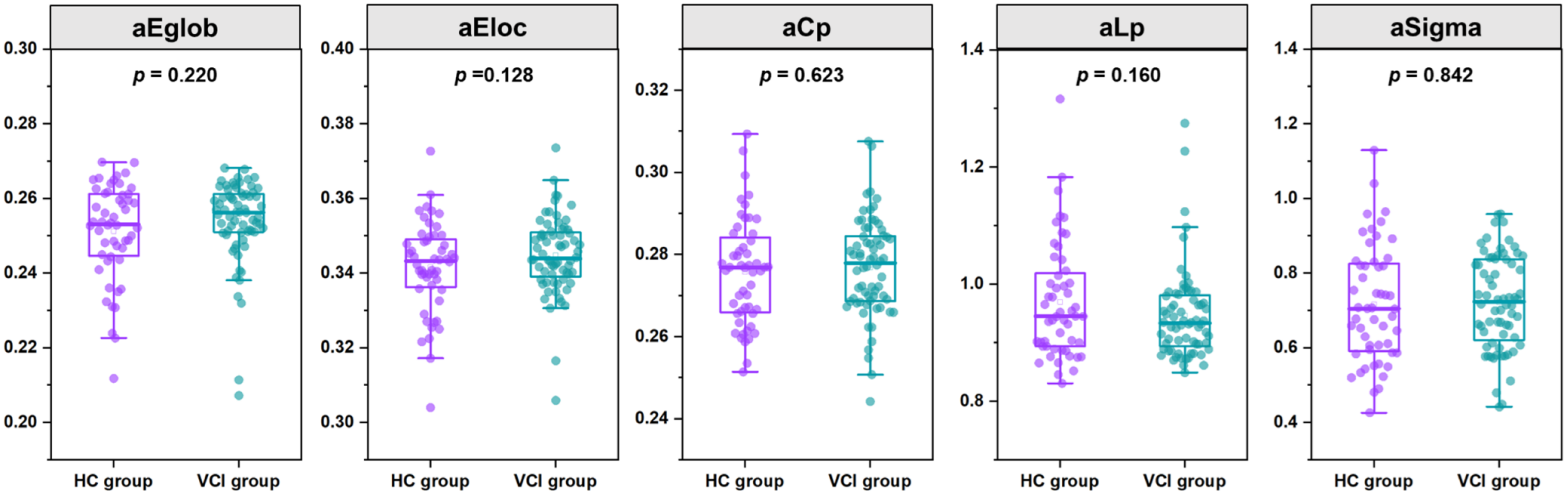
Group differences in functional network topological properties between vascular cognitive impairment (VCI) participants and healthy controls (HCs). Abbreviations: aCp, area under the curve for clustering coefficient; AEglob, area under the curve for global efficiency; aEloc, area under the curve for local efficiency; aLp, area under the curve for characteristic path length; aSigma, area under the curve for sigma.

The analysis of functional network topological properties showed no statistically significant differences between VCI participants and HCs in global efficiency, local efficiency, clustering coefficient, characteristic path length, and small‐worldness (Sigma) (Figure 4). Additionally, intergroup comparisons revealed no nodal property differences after FWE correction.

### 3.5 Structure–Function Coupling

**FIGURE 5:**
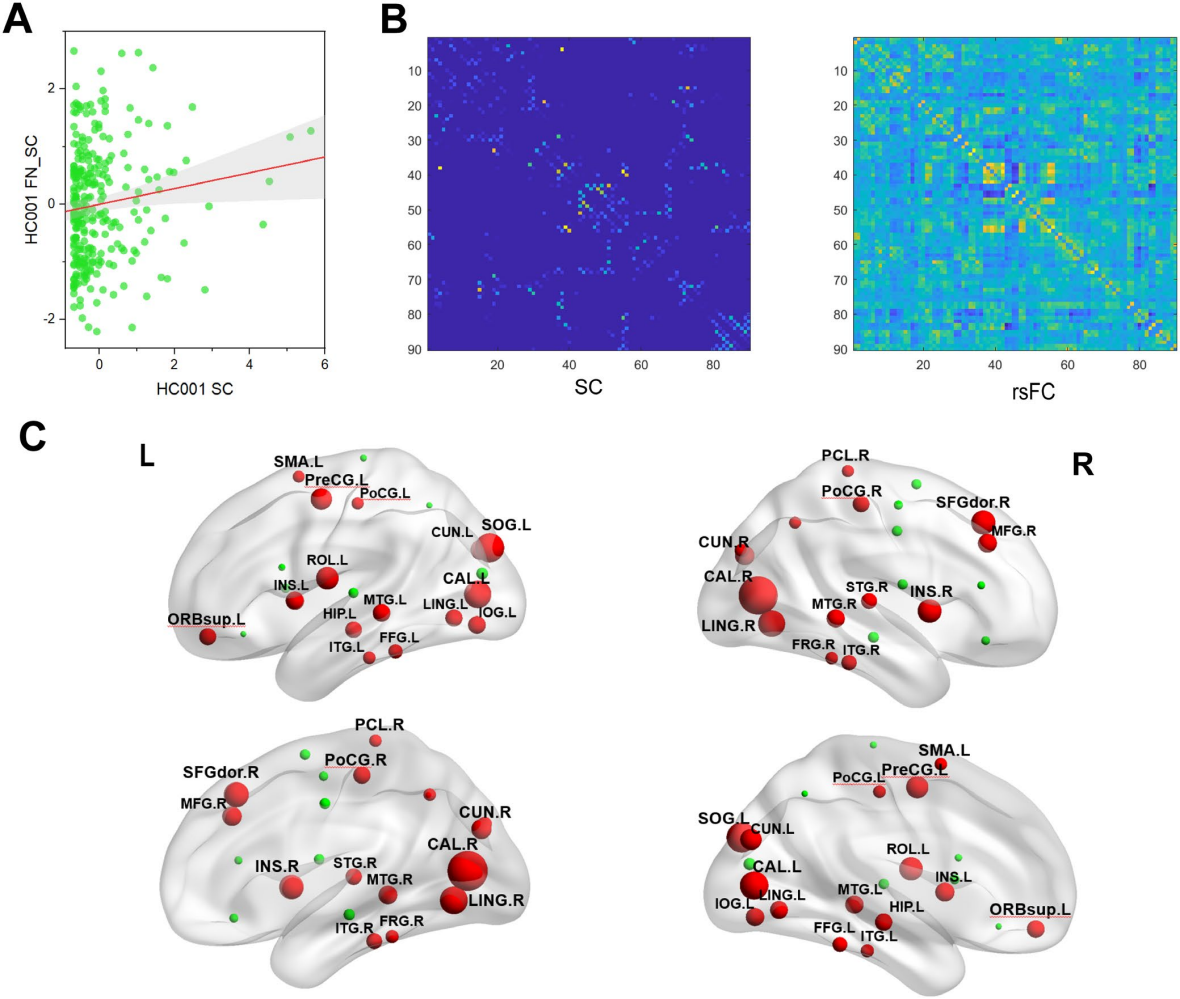
Structure–function coupling. (A) Scatter plot of resting‐state functional connectivity (rsFC) against structural connectivity (SC) at high resolution for healthy control 001 (HC 001), showing edges with nonzero SC. (B) Sparse structural brain network connectivity map and resting‐state functional network connectivity map for HC participant 001 (sparsity 0.01–0.4, step 0.01). (C) Insignificant nodes are shown as green spheres, while red spheres (VCI>HC) denote significant differences after FWE correction, with regions labeled where *p* < 0.0001. Abbreviations: CAL, calcarine cortex; CUN, cuneus; FFG, fusiform gyrus; HIP, hippocampus; INS, insula; IOG, inferior occipital gyrus; ITG, inferior temporal gyrus; L, left; LING, lingual gyrus; MTG, middle temporal gyrus; PCUN, right precuneus; STG, superior temporal gyrus; MFG, middle frontal gyrus; ORBsup, superior orbital gyrus, PCL, paracentral lobule; PoCG, postcentral gyrus; PreCG, precentral gyrus; R, right; SFGdor, dorsolateral superior frontal gyrus; SMA, supplementary motor area; SOG, superior occipital gyrus.

A positive correlation trend between the FA structural and resting‐state functional matrices of participant HC 001 was unfolded into vectors (Figure 5A). This trend was also evident in participant HC 001's network graph, where regions of interest (ROIs) with structural connectivity showed strong functional connectivity (Figure 5B).

Group comparisons of nodal coupling relationships revealed that VCI participants had higher structure–function coupling in bilateral calcarine cortex, bilateral cuneus, bilateral superior occipital gyrus, bilateral lingual gyrus, bilateral insula, bilateral middle temporal gyrus, bilateral postcentral gyrus, bilateral inferior temporal gyrus, bilateral fusiform gyrus, right middle frontal gyrus, left inferior occipital gyrus, left superior orbital gyrus, left hippocampus, right superior temporal gyrus, right dorsolateral superior frontal gyrus, left supplementary motor area, right paracentral lobule, right precuneus, left precentral gyrus, and left Rolandic operculum compared to HCs (*p* < 0.0001, FWE‐corrected) (Figure 5C).

### 3.6 The Impact of Structure–Function Coupling in Cognitive Function

**FIGURE 6:**
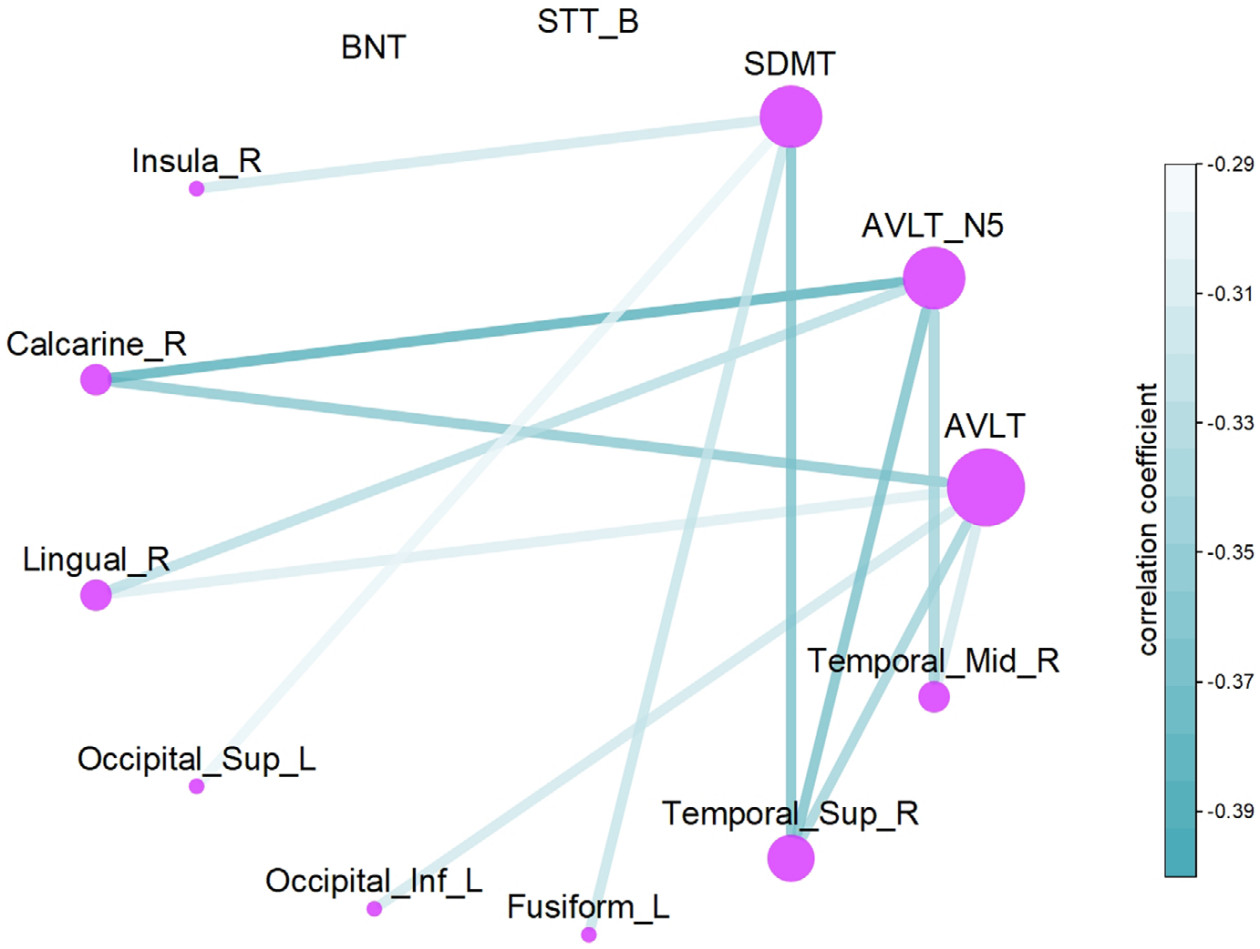
Circos diagram of structural–functional coupling and cognitive function. The color intensity of the lines represents the correlation coefficient value, with darker lines indicating a stronger correlation between the brain region and the cognitive function at the respective node. The size of the node's circle corresponds to the number of connections, indicating the degree of influence of the node. Abbreviations: AVLT, Auditory Verbal Learning Test; AVLT_N5, Auditory Verbal Learning Test, Long‐Term Delay Recall; BNT, Boston Naming Test; Inf, inferior; L, left; Mid, middle; R, right; SDMT correct, Symbol Digit Modalities Test, correct number; STT_B, Shape Trails Test, part B; Sup, Superior.

To investigate the increased structural–functional coupling in VCI patients compared to HCs, partial correlation analyses were conducted to assess the relationship between structural–functional coupling in differing regions and performance on the BNT, STT_B, SDMT, and AVLT (Figure 6). The results showed a significant negative correlation between coupling in the Calcarine_R, Lingual_R, Occipital_Inf_L, Fusiform_L, Temporal_Sup_R, and Temporal_Mid_R regions and AVLT scores, which reflects memory function (*p* < 0.001, FWE‐corrected). Additionally, a significant negative correlation was observed between coupling in the Insula_R, Calcarine_R, Lingual_R, Occipital_Sup_L, Occipital_Inf_L, Fusiform_L, and Temporal_Sup_R regions and SDMT scores, reflecting information processing speed (*p* < 0.001, FWE‐corrected). The uncorrected outcomes are detailed in the Supporting Informations.

## 4 Discussion

Building on previous research and our formulated hypothesis, our findings confirm that participants with VCI exhibit impaired brain fiber integrity compared to HCs. In terms of structural brain network topology, VCI participants showed reductions in global efficiency, local efficiency, shortest path length, and small‐world parameters (Sigma). Although the clustering coefficient did not show a statistically significant difference, a decreasing trend was also observed. To investigate certain inconsistent nodes, we found decreased node connectivity in the white matter structural network of VCI participants, particularly in regions associated with the default mode network (DMN) and the visual network (VN). Contrary to our hypothesis, no significant differences in functional brain network topology were found between VCI participants and HCs. However, in terms of structure–function coupling, VCI participants showed significantly higher structure–function coupling in several brain regions. Partial correlation analyses of the structural–functional coupling in different brain regions with cognitive scores revealed that the Calcarine, Lingual, Occipital, Fusiform, and Temporal regions exhibited a negative correlation with memory function. Additionally, a significant negative correlation was observed between coupling in the Insula, Calcarine, Lingual, Occipital, Fusiform, and Temporal regions and information processing speed.

DTI measures the directional diffusivity of water, thereby revealing the breakdown of tissue microarchitecture or fluid shifts between the intracellular, extracellular, or vascular spaces [40, 41]. Conventional DTI metrics, including MD and FA, are altered in chronic cerebrovascular disease even in normal appearing white matter [42] and are associated with cognitive performance [43, 44, 45]. Chronic white matter injury manifests as increased MD and decreased FA, MD, and RD. Our results indicated that white matter integrity was compromised in participants with VCI. Specifically, our study observed an increase in fractional anisotropy, coupled with increases in MD and RD, indicating disrupted white matter microstructure in VCI participants. These alterations in DTI metrics suggest myelin loss and axonal damage, likely resulting from ischemic changes, inflammation, and oxidative stress. Previous investigations have revealed a decrease in the global properties of structural networks among individuals with cognitive impairments [46]. Consistently, our study has also observed a decline in global efficiency, local efficiency, shortest path length, and small‐world parameters within the white matter structural networks of VCI participants. This finding underscores the compromised connectivity and integration within the brain's structural networks in VCI, which may reflect alterations in the underlying microstructural integrity and axonal connectivity.

To investigate certain inconsistent nodes, we conducted an analysis of node properties. The left posterior cingulate gyrus, right precuneus, and right posterior cingulate gyrus are key nodes in the DMN, which is involved in self‐referential processing and memory consolidation [47, 48]. The observed decreases in degree centrality and nodal efficiency suggest reduced connectivity and information processing efficiency within the DMN, potentially leading to cognitive deficits associated with VCI. Furthermore, the increase in the nodal shortest path in regions such as the left posterior cingulate gyrus, right precuneus, and left precuneus indicates a longer average distance for information to travel between nodes, which can affect the speed and efficiency of cognitive processing. Specific regions such as posterior cingulate gyrus, right posterior cingulate gyrus, right middle occipital gyrus, and right cuneus exhibited structural alterations in VCI ^[^ 19]. The involvement of posterior cingulate and precuneus regions suggests impairments in attentional networks and self‐directed processing [49], whereas changes in visual processing areas such as right middle occipital gyrus and right cuneus may affect visual information processing and attentional mechanisms [50].

After identifying structural network topological differences, we further examined functional network topological properties but found no significant group differences. This lack of significant differences in functional network topology between VCI patients and control subjects could be due to several factors. It is possible that the functional networks exhibit compensatory mechanisms that mask the underlying structural deficits or that the sensitivity of the functional connectivity measures used in our study may not have been sufficient to detect subtle changes in the early stages of VCI.

However, in structure–function coupling, VCI participants exhibited significantly higher coupling in several brain regions, including the bilateral calcarine cortex, cuneus, superior occipital gyrus, lingual gyrus, insula, middle temporal gyrus, postcentral gyrus, inferior temporal gyrus, fusiform gyrus, right middle frontal gyrus, left inferior occipital gyrus, left superior orbital gyrus, left hippocampus, right superior temporal gyrus, right dorsolateral superior frontal gyrus, left supplementary motor area, right paracentral lobule, right precuneus, left precentral gyrus, and left Rolandic operculum. Additionally, the degree of structural–functional coupling in the temporal calcarine insula, occipital, and lingual regions was negatively associated with memory function and information processing speed. This suggests a compensatory increase in structure–function coupling in VCI to maintain memory function and information processing speed. The increase in structure–function coupling could be a mechanism by which the brain attempts to adapt to the loss of structural integrity, by increasing functional efficiency in regions that are still relatively intact [51]. Alternatively, the higher structure–function coupling might also indicate a pathological adaptation, where the brain is making inefficient use of its remaining resources, leading to an overcompensation that is not necessarily beneficial in the long term. This could be a sign of the brain's attempt to maintain function in the face of structural damage, which might ultimately result in a maladaptive state that contributes to the progression of the cognitive decline in VCI patients. The regional specificity of the increased structure–function coupling in VCI suggests that these areas might be particularly vulnerable or play a key role in memory function and information processing speed. Future studies could explore whether these regions could serve as biomarkers for early detection or as targets for therapeutic interventions in VCI. Understanding the underlying mechanisms of increased structure–function coupling could provide valuable insights into the cognitive function and help guide the development of more effective treatment strategies.

### 4.1 Limitations

This study has several limitations. Despite employing multiple neuroimaging techniques, the cross‐sectional design prevents establishing causality between white matter integrity and cognitive impairment in VCI. Additionally, the reliance on cognitive scales and Fazekas scores for classification might not comprehensively reflect the spectrum of cognitive decline or white matter changes. Potential head motion during MRI scanning may compromise the accuracy of DTI metrics. Moreover, the lack of significant differences in functional network connectivity analysis might indicate limitations in methodological sensitivity. Future research should include larger, longitudinal studies to validate and extend these findings.

### 4.2 Conclusions

This study identified notable neurocognitive and brain connectivity differences between individuals with VCI and HCs. VCI participants exhibited poorer cognitive performance and significant disruptions in white matter integrity, evidenced by decreased FA and increased MD and RD. Structural network analysis indicated reduced global and local efficiency, diminished small‐world properties, along with altered nodal connectivity in several brain regions. Additionally, the differences in structural–functional coupling in these brain regions were negatively correlated with memory function and information processing speed. These findings highlight the extensive neural alterations in VCI and underscore the importance of integrating structural and functional connectivity analyses to understand the mechanisms of cognitive impairment.

## Conflicts of Interest

The authors declare no conflicts of interest.

## Transparency Statement

This observational case–control study adheres to the principles of transparency and reproducibility. The study design, data collection procedures, and statistical analysis methods are clearly described in the manuscript. Ethical approval was obtained for this study, and informed consent was acquired from all participants. No changes to the study design or data analysis plan were made after the commencement of the study.

## Supporting information


Data S1.

## Data Availability

The data that support the findings of this study are available on request from the corresponding author. The data are not publicly available due to privacy or ethical restrictions.
