# Supplementary material for: Neural Correlates and Adaptive Mechanisms in Vascular Cognitive Impairment: Exploration of a Structure–Function Coupling Network
Source: CNS Neurosci Ther. 2025 Mar 9;31(3):e70205. doi: 10.1111/cns.70205 (PMC11890977; doi:10.1111/cns.70205)
Supplement: Supplementary file 1 — Data S1. [file CNS-31-e70205-s001.docx]

***Supplementary Materials***

**for the article**

**“Neural Correlates and Adaptive Mechanisms in Vascular Cognitive Impairment: An Exploration of Structure-Function Coupling Network”**

**Supplementary Methods: Page 2 – 5**

**Supplementary Results: Page 6**

**Supplementary References: Page 7**

### The calculation formula of each parameter in this study

***Average Path Length.*** For a binary network, the shortest path is the path with the least number of edges connecting two nodes, and the number of edges in the path is the shortest path length between the two nodes. It describes the best path from one node in the network to another node. Through the shortest path, information can be transmitted faster, thus saving system resources. It reflects the efficiency of long-distance communication in the brain. By averaging the shortest path length between any two nodes in the network, the characteristic path length L of the entire network can be obtained.

$$L(G)=\frac{1}{N(N-1)}\sum_{i,j,i\neq j\in G} d_{i,j}$$

Where N represents the total number of nodes within the network $G$, while $d_{i,j}$ denotes the distance between node $i$ and node $j$in the network $G$.

***Global efficiency.*** Global efficiency measures the parallel information transfer ability of the network, which can be computed as follows:

$$E_{global}(G)=\frac{1}{N(N-1)}\sum_{i,j,i\neq j\in G} \frac{1}{d_{ij}}$$

Where N represents the total number of nodes within the network $G$, while $d_{i,j}$ denotes the distance between node $i$ and node $j$in the network $G$.

***Local efficiency.*** Local efficiency measures the information exchanging ability among a sub-graph with locally interconnected nodes. It reflects system redundancy and tolerance to attack, which can be computed as follows:

$$E_{loc}(G)=\frac{1}{N}\sum_{i\in G} Eglobal(G_{i})$$

where N is the number of nodes in the network $G$, and $G_{i}$ is the sub-graph consisting of node $i$ and its local neighbors.

***Average clustering coefficient.*** The clustering coefficient $C_{i}$ was defined as the fraction of the neighbors of a node $i$ that are also neighbors of each other^9^ and was chosen to reflect local efficiency because it generally conveys similar information as Eloc; it can be described as:

$$C_{i}(G)=\frac{E_{i}}{\frac{1}{2}k_{i}(k_{i}-1)}$$

Where $C_{i}$ represents the node $i$’s weighted clustering coefficient. $k_{i}$ was the node $i$'s degree, and $E_{i}$ was the geometric mean of triangles in the vicinity of $i$.

***Small world parameters.*** Small-world networks, which are between regular networks and random networks, have high clustering coefficient and short feature path length. The small-world parameter σ is used to describe the strength of the small-world attribute of the network. σ > 1 indicates that the network has small-world attribute. The larger the σ is, the stronger the small-world attribute will be. The small-world parameter calculation formula is as follows:

$$\sigma=\frac{\gamma}{\lambda}$$

Where $\gamma=\frac{C_{real}}{C_{random}}\gg1$, $\lambda=\frac{L_{real}}{L_{random}}\approx1$. The subscript real denotes a computation network and random denotes a random network.

***Nodal local efficiency***. The nodal local efficiency of node $i$ is defined in the subgraph of the direct neighbors of $i$:

$${nodalE}_{loc}(G,i)=\frac{1}{N_{Gi}(N_{Gi}-1)}\sum_{i,j,i\neq k\notin Gi} \frac{1}{d_{ik}}$$

where $N_{Gi}$ is the number of nodes in the subgraph $Gi$ consisting of all of the neighbors of $i$; and $d_{jk}$ is the length of the shortest path, in terms of tractography-based measures of physical distances, between nodes $j$ and $k$.

***Nodal global efficiency.*** The nodal global efficiency of node $i$ is computed as:

$${nodalE}_{glob}(G,i)=\frac{1}{N(N-1)}\sum_{i,j,i\neq j∊G} \frac{1}{d_{ij}}$$

where $N$ is the number of nodes in the network graph $G$; and $d_{ij}$ is the length of the shortest path, in terms of tractography-based measures of physical distances, between nodes $i$ and $j$.

***Nodal centrality***. Degree $k_{i}$was used to measure the centrality of a node. It is defined as the number of links connected to the node, and it is a simple measurement of connectivity of a node with the rest of nodes in a network. Hub regions often interact with many other regions in the network and thus have high centrality. Formally, in a network $G$ with $N$ nodes and $k$ edges, the degree $k_{i}$ of node $i$ is defined as:

$$k_{i}=\sum_{j∊G} a_{ij}$$

where $a_{ij}$ is the $i$ th row and $j$ th column element of the adjacency matrix.

***MRI Preprocessing***

Firstly, converting the original digital imaging and communication in medicine (DICOM) image into neuroimaging informatics technology initiative (NIfTI) format.

Preprocessing of BOLD imaging data was conducted using the Statistical Parametric Mapping toolbox (SPM12, http://www.fil.ion.ucl.ac.uk/spm) within MATLAB R2022b (MathWorks Inc., Natick, MA, USA). The initial 10 volumes of each participant's dataset were discarded. The remaining 190 images were aligned with anatomical datasets using the anterior commissure as a reference. Participants exhibiting head movements exceeding 3.0 mm or rotations over 3.0° were excluded. The functional images were initially normalized to the standard brain using T1-weighted images and then spatially aligned to the Montreal Neurological Institute (MNI) space with pre-estimated deformation parameters, before being resampled to 3 mm cubic voxels. Finally, the data was smoothed using a 6 mm full width half maximum Gaussian kernel to increase BOLD signal to noise‐ratio. Both the BOLD-weighted time series and the artefactual model time series were temporally filtered with a passband between 0.01 and 0.08 Hz^[1]^.

Preprocessing of DTI imaging data was conducted using the FMRIB Software Library (FSL, <http://www.fmrib.ox.ac.uk/fsl>, version 5.0.9) and MRtrix3 (<https://www.mrtrix.org/>, version 3.0)^[2]^. Initially, data in NIfTI format was converted to MRtrix3 image format (MIF). This conversion was followed by sequential denoising and the removal of Gibbs ringing artifacts. Subsequent steps included correction for head motion and eddy current distortions^[3]^. The Brain Extraction Tool (BET) was employed to excise non-brain tissue^[4]^. Finally, after performing bias field correction, the data was converted back from MIF to NIfTI format. All data underwent manual inspection for the accuracy of skull stripping and registration precision.

**sTable1 Structural-Functional Coupling and Cognitive Function**

|  | AVLT | | AVLT_N5 | | SDMT | | STT_B | | BNT | | HDMD | |
| --- | --- | --- | --- | --- | --- | --- | --- | --- | --- | --- | --- | --- |
|  | Corr. | Sig. | Corr. | Sig. | Corr. | Sig. | Corr. | Sig. | Corr. | Sig. | Corr. | Sig. |
| Precentral_L | -0.216 | 0.019 | -0.225 | 0.015 | -0.146 | 0.116 | 0.138 | 0.137 | -0.264 | 0.004 | -0.091 | 0.329 |
| Precentral_R | -0.139 | 0.136 | -0.152 | 0.102 | -0.127 | 0.171 | 0.112 | 0.228 | -0.181 | 0.051 | -0.213 | 0.021 |
| Frontal_Sup_R | -0.281 | 0.002 | -0.247 | 0.007 | -0.221 | 0.016 | 0.289 | 0.002 | -0.080 | 0.391 | -0.034 | 0.714 |
| Frontal_Sup_Orb_L | -0.178 | 0.055 | -0.177 | 0.057 | -0.245 | 0.008 | 0.096 | 0.302 | -0.190 | 0.040 | -0.230 | 0.012 |
| Frontal_Mid_R | -0.243 | 0.008 | -0.231 | 0.012 | -0.128 | 0.170 | 0.250 | 0.007 | -0.121 | 0.194 | -0.203 | 0.028 |
| Frontal_Inf_Oper_L | -0.109 | 0.243 | -0.160 | 0.084 | -0.210 | 0.023 | 0.147 | 0.114 | -0.034 | 0.719 | 0.012 | 0.900 |
| Frontal_Inf_Tri_R | -0.141 | 0.130 | -0.150 | 0.107 | -0.072 | 0.441 | 0.042 | 0.652 | -0.164 | 0.077 | -0.036 | 0.697 |
| Frontal_Inf_Orb_L | -0.017 | 0.853 | -0.035 | 0.710 | -0.120 | 0.197 | 0.100 | 0.281 | -0.154 | 0.098 | 0.119 | 0.203 |
| Frontal_Inf_Orb_R | -0.215 | 0.020 | -0.242 | 0.008 | 0.032 | 0.730 | -0.025 | 0.786 | -0.084 | 0.367 | -0.182 | 0.050 |
| Rolandic_Oper_L | -0.257 | 0.005 | -0.287 | 0.002 | -0.130 | 0.162 | 0.146 | 0.116 | -0.141 | 0.129 | -0.029 | 0.758 |
| Rolandic_Oper_R | -0.235 | 0.011 | -0.212 | 0.022 | -0.175 | 0.059 | 0.176 | 0.057 | -0.111 | 0.234 | 0.125 | 0.179 |
| Supp_Motor_Area_L | -0.127 | 0.171 | -0.186 | 0.045 | -0.119 | 0.203 | 0.085 | 0.362 | -0.043 | 0.647 | -0.190 | 0.041 |
| Supp_Motor_Area_R | -0.170 | 0.066 | -0.165 | 0.076 | -0.065 | 0.485 | 0.119 | 0.201 | 0.026 | 0.779 | -0.020 | 0.828 |
| Insula_L | -0.288 | 0.002 | -0.292 | 0.001 | -0.230 | 0.013 | 0.170 | 0.067 | -0.092 | 0.322 | -0.229 | 0.013 |
| Insula_R | -0.274 | 0.003 | -0.255 | 0.005 | -0.315 | 0.001 | 0.282 | 0.002 | -0.147 | 0.115 | -0.119 | 0.202 |
| Cingulum_Mid_R | -0.106 | 0.256 | -0.079 | 0.397 | 0.047 | 0.612 | 0.187 | 0.043 | -0.179 | 0.053 | -0.049 | 0.603 |
| Hippocampus_L | -0.290 | 0.002 | -0.265 | 0.004 | -0.194 | 0.036 | 0.149 | 0.110 | -0.094 | 0.312 | -0.009 | 0.922 |
| Hippocampus_R | -0.167 | 0.071 | -0.148 | 0.112 | -0.215 | 0.020 | 0.248 | 0.007 | -0.103 | 0.270 | -0.027 | 0.770 |
| Calcarine_L | -0.254 | 0.006 | -0.256 | 0.005 | -0.265 | 0.004 | 0.262 | 0.004 | -0.288 | 0.002 | -0.141 | 0.130 |
| Calcarine_R | -0.358 | 0.000 | -0.387 | 0.000 | -0.296 | 0.001 | 0.194 | 0.036 | -0.273 | 0.003 | -0.099 | 0.290 |
| Cuneus_L | -0.294 | 0.001 | -0.212 | 0.022 | -0.244 | 0.008 | 0.236 | 0.010 | -0.105 | 0.259 | -0.084 | 0.370 |
| Cuneus_R | -0.118 | 0.205 | -0.121 | 0.195 | -0.209 | 0.024 | 0.178 | 0.055 | -0.137 | 0.140 | -0.001 | 0.994 |
| Lingual_L | -0.233 | 0.011 | -0.226 | 0.014 | -0.193 | 0.037 | 0.152 | 0.103 | -0.239 | 0.009 | -0.159 | 0.087 |
| Lingual_R | -0.310 | 0.001 | -0.331 | 0.000 | -0.159 | 0.087 | 0.116 | 0.212 | -0.253 | 0.006 | 0.038 | 0.682 |
| Occipital_Sup_L | -0.243 | 0.008 | -0.282 | 0.002 | -0.302 | 0.001 | 0.223 | 0.016 | -0.127 | 0.173 | -0.206 | 0.026 |
| Occipital_Sup_R | -0.290 | 0.002 | -0.276 | 0.003 | -0.240 | 0.009 | 0.086 | 0.358 | -0.216 | 0.019 | -0.144 | 0.121 |
| Occipital_Mid_L | -0.135 | 0.148 | -0.142 | 0.127 | -0.163 | 0.080 | 0.120 | 0.197 | -0.110 | 0.238 | -0.062 | 0.504 |
| Occipital_Inf_L | -0.313 | 0.001 | -0.295 | 0.001 | -0.186 | 0.045 | 0.079 | 0.396 | -0.133 | 0.152 | -0.092 | 0.323 |
| Fusiform_L | -0.230 | 0.013 | -0.224 | 0.015 | -0.322 | 0.000 | 0.182 | 0.050 | -0.137 | 0.142 | -0.075 | 0.424 |
| Fusiform_R | -0.220 | 0.017 | -0.213 | 0.021 | -0.199 | 0.031 | 0.177 | 0.057 | -0.185 | 0.046 | -0.171 | 0.066 |
| Postcentral_L | -0.192 | 0.038 | -0.223 | 0.016 | -0.257 | 0.005 | 0.241 | 0.009 | -0.244 | 0.008 | -0.148 | 0.110 |
| Postcentral_R | -0.243 | 0.008 | -0.269 | 0.003 | -0.247 | 0.007 | 0.150 | 0.105 | -0.024 | 0.793 | -0.115 | 0.219 |
| Precuneus_L | 0.025 | 0.792 | 0.015 | 0.869 | -0.019 | 0.843 | -0.016 | 0.867 | 0.073 | 0.432 | -0.035 | 0.711 |
| Precuneus_R | -0.151 | 0.105 | -0.148 | 0.111 | -0.123 | 0.185 | 0.136 | 0.144 | -0.124 | 0.181 | -0.143 | 0.125 |
| Paracentral_Lobule_L | -0.085 | 0.360 | -0.078 | 0.404 | -0.062 | 0.507 | 0.215 | 0.020 | 0.149 | 0.110 | -0.097 | 0.300 |
| Paracentral_Lobule_R | -0.066 | 0.480 | -0.078 | 0.403 | -0.059 | 0.527 | 0.105 | 0.258 | -0.105 | 0.262 | -0.115 | 0.218 |
| Caudate_L | -0.284 | 0.002 | -0.290 | 0.002 | -0.236 | 0.010 | 0.152 | 0.101 | -0.196 | 0.034 | -0.181 | 0.050 |
| Temporal_Sup_L | -0.129 | 0.166 | -0.127 | 0.172 | -0.111 | 0.235 | 0.086 | 0.358 | -0.078 | 0.405 | 0.154 | 0.097 |
| Temporal_Sup_R | -0.346 | 0.000 | -0.366 | 0.000 | -0.365 | 0.000 | 0.230 | 0.012 | -0.160 | 0.085 | -0.032 | 0.728 |
| Temporal_Mid_L | -0.263 | 0.004 | -0.276 | 0.003 | -0.124 | 0.184 | 0.146 | 0.118 | -0.243 | 0.008 | -0.039 | 0.672 |
| Temporal_Mid_R | -0.314 | 0.001 | -0.347 | 0.000 | -0.216 | 0.019 | 0.155 | 0.095 | -0.202 | 0.029 | -0.185 | 0.046 |
| Temporal_Inf_L | -0.235 | 0.011 | -0.192 | 0.038 | -0.196 | 0.034 | 0.243 | 0.008 | -0.224 | 0.015 | 0.046 | 0.620 |
| Temporal_Inf_R | -0.294 | 0.001 | -0.260 | 0.005 | -0.153 | 0.100 | 0.155 | 0.095 | -0.169 | 0.068 | 0.012 | 0.898 |

**Supplementary References**

1. Hallquist MN, Hwang K, Luna B. The nuisance of nuisance regression: spectral misspecification in a common approach to resting-state fMRI preprocessing reintroduces noise and obscures functional connectivity. Neuroimage. 2013;82:208-25. doi: 10.1016/j.neuroimage.2013.05.116.

2. Tournier JD, Smith R, Raffelt D, Tabbara R, Dhollander T, Pietsch M, et al. MRtrix3: A fast, flexible and open software framework for medical image processing and visualisation. Neuroimage. 2019;202:116137. doi: 10.1016/j.neuroimage.2019.116137.

3. Jones DK, Cercignani M. Twenty-five pitfalls in the analysis of diffusion MRI data. NMR Biomed. 2010;23(7):803-20. doi: 10.1002/nbm.1543.

4. Smith SM. Fast robust automated brain extraction. Hum Brain Mapp. 2002;17(3):143-55. doi: 10.1002/hbm.10062.
